# Hip abduction angle after open-wedge high tibial osteotomy is associated with the timed up & go test and recurrence of varus alignment

**DOI:** 10.1038/s41598-023-33481-9

**Published:** 2023-04-29

**Authors:** Youngji Kim, Mitsuaki Kubota, Taisuke Sato, Hiroki Tanabe, Ryuichi Ohno, Muneaki Ishijima

**Affiliations:** 1 Department of Orthopaedic Surgery, Koshigaya Municipal Hospital, 3-1-3 Hongo, Bunkyoku, Tokyo, 113-8431 Japan; 2 Department of Orthopaedic Surgery and Sports Medicine, Faculty of Medicine, Juntendo University School of Medicine, 3-1-3 Hongo, Bunkyoku, Tokyo, 113-8431 Japan

**Keywords:** Medical research, Risk factors

## Abstract

The aim of this study is to investigate the association between the hip abduction angle (HAA) and lower limb alignment as well as the clinical assessments in open-wedge high tibial osteotomy (OWHTO) patients. A total of 90 patients who underwent OWHTO were included. The demographic characteristics and clinical assessments (the Visual Analogue Scale for activities of daily living, the Japanese knee osteoarthritis measure, the Knee injury and Osteoarthritis Outcome Score, the Knee Society score, the Timed Up & Go (TUG) test, the single standing (SLS) test and muscle strength) were recorded. The patients were divided into two groups according to the HAA at 1 month after operation: the HAA (−) group (HAA < 0°) and the HAA (+) group (HAA ≥ 0°). Clinical scores except for the SLS test and radiographic parameters except for the posterior tibia slope (PTS), lateral distal femoral angle (LDFA) and lateral distal tibial angle (LDTA) were significantly improved at 2 years postoperatively. Regarding the two groups, scores on the TUG test in the HAA (−) group were significantly lower than those in the HAA (+) group (p = 0.011). The hip-knee-ankle angle (HKA), weight bearing line (WBLR) and knee joint line obliquity (KJLO) in the HAA (−) group were significantly higher than those in the HAA (+) group (p < 0.001, 0.001 and p = 0.025). In contrast, the LDFA in the HAA (−) group were significantly lower than those in the HAA (+) group (p < 0.001). The TUG test and the LDFA were weakly positively correlated with the HAA (r = 0.34, 0.42, p < 0.001 and 0.001). In contrast, the HKA, WBLR and KJLO had a weak negative correlation with the HAA (r = − 0.43, − 0.38 and − 0.37, p < 0.001, 0.001 and 0.001). This study showed the postoperative HAA was significantly associated with the TUG test and the HKA, WBLR, LDFA, and KJLO. A higher postoperative HAA might induce varus recurrence and poor outcomes of the gait parameter.

## Introduction

Open-wedge high tibial osteotomy (OWHTO) is commonly conducted to preserve the knee joint and to correct alignment for medial compartment knee osteoarthritis^1–3^. This operation is an effective surgery with positive long-term results if corrections of the medial tibial angle and lower extremity mechanical alignment are appropriate by passing the mechanical alignment through Fujisawa point^4,5^.

The hip joint is important for knee joint kinematics^6^. The hip abductor muscle contributes to single-leg standing and walking by stabilizing pelvic tilt. In patients with medial compartment knee osteoarthritis, hip abductor muscle weakness induces pelvic tilt and leads to medial compartment loading^7^.

There are some reports of a relationship between the alignment of the lower limb after OWHTO and ankle alignments, such as Tibial plafond inclination (TPI), talar inclination (TI), talar tilt (TT), and lateral distal tibial angle (LDTA)^8–11^. However, few reports have focused on hip joint alignment after OWHTO. The outcome of OWHTO patients with an abnormality of hip joint alignment is uncertain, and data related to the association between hip abduction angle and clinical and functional scores are scarce.

The main objective of this study was to investigate the association between the postoperative hip abduction angle (HAA) and radiological parameters of lower limb alignment as well as the clinical and functional scores in OWHTO patients. The hypothesis is that a higher HAA may be associated with varus alignment and poor clinical and functional outcomes.

## Results

### Patient demographic data

The mean age was 61.2 ± 9.9 years, height was 159.8 ± 7.3 cm, weight was 68.2 ± 12.3 kg, and body mass index (BMI) was 26.6 ± 3.6 kg/m^2^. There were 30 males (33.3%) and 60 females (66.7%). The affected joint side was the right knee in 56 (62.2%) patients and the left knee in 34 (37.8%) patients. The mean correction angle was 12.1 ± 3.3°. In addition, there were no significant differences in demographic data between the two groups. The patients’ demographic data are summarized in Table 1.

**Table 1:** Patient demographic.

Variable	All		HAA (−) (n = 52)		HAA (+) (n = 38)		p-value	
Age (years)	61.2 ± 9.9		60.3 ± 10.1		62.5 ± 9.8		0.33	
Height (cm)	159.8 ± 7.3		160.9 ± 7.6		145.0 ± 6.8		0.14	
Weight (kg)	68.2 ± 12.3		68.0 ± 12.1		68.5 ± 12.6		0.89	
BMI (kg/m^2^)	26.6 ± 3.6		26.1 ± 3.5		27.2 ± 3.6		0.27	
Sex	Male	30	33.3%	16	34.2%	13	23.7%	0.77
	Female	60	66.7%	36	65.8%	25	76.3%	
Affected joint side	Right	56	62.2%	21	34.2%	13	44.7%	0.86
	Left	34	37.8%	31	65.8%	25	55.3%	
Correction angle (°)	12.1 ± 3.3		11.5 ± 3.1		12.9 ± 3.4		0.04*	

Data are shown as the means with standard deviation or number.

*BMI* body mass index.

### Clinical and functional scores and radiographic data preoperatively and 2 years postoperatively

No clinical and functional scores were significantly different, in radiological parameters, HKA and WBLR were significantly different at preoperatively between the HAA (−) group and the HAA (+) group. (Supplemental Table S1).

Clinical and functional scores were improved at 2 years postoperatively compared to preoperatively, except for the SLS. Additionally, most radiographic parameters were improved at 2 years postoperatively compared to preoperatively. The PTS, LDFA and LDTA were not changed at 2 years postoperatively compared to preoperatively (Table 2).

**Table 2:** Clinical and functional score and radiographic data at pre-opeartive and post-operative 2 year.

	Pre-	Post-operative 2 year	*p-*value
Clinical score			
JKOM			
VAS for ADL	60.4 ± 25.5	16.5 ± 21.1	< 0.001*
Pain and stiffness	16.4 ± 6.7	5.4 ± 4.9	< 0.001*
ADL	13.1 ± 6.9	5.1 ± 5.5	< 0.001*
Activities	8.4 ± 5.7	3.8 ± 4.2	< 0.001*
Health condition	3.6 ± 2.1	2.0 ± 1.8	< 0.001*
Total	41.7 ± 18.1	16.3 ± 14.4	< 0.001*
KOOS			
Symptom	59.2 ± 18.9	81.8 ± 15.3	< 0.001*
Pain	49.9 ± 18.6	80.6 ± 15.9	< 0.001*
ADL	66.1 ± 16.3	87.1 ± 12.7	< 0.001*
Sports	30.0 ± 19.8	60.6 ± 26.0	< 0.001*
QOL	28.1 ± 15.2	63.7 ± 24.7	< 0.001*
KSS	66.4 ± 6.3	92.1 ± 7.7	< 0.001*
TUG, s	9.8 ± 2.6	8.0 ± 1.9	< 0.001*
SLS, s	19.4 ± 11.8	21.8 ± 10.9	0.07
Isometric muscle strength (%BW)			
Quadriceps	108.8 ± 46.5	135.9 ± 42.0	< 0.001*
Hamstring	53.0 ± 27.6	79.5 ± 33.6	< 0.001*
Radiological parameters			
HAA, °	2.2 ± 2.8	− 1.0 ± 3.1	< 0.001*
HKA, °	− 7.2 ± 3.5	2.7 ± 3.7	< 0.001*
LDFA, °	88.7 ± 1.9	88.3 ± 2.2	0.187
MPTA, °	84.9 ± 2.5	92.7 ± 3.1	< 0.001*
WBLR, %	16.4 ± 12.7	56.4 ± 15.3	< 0.001*
JLCA, °	3.0 ± 2.0	2.3 ± 2.0	< 0.001*
KJLO, °	0.4 ± 3.1	2.6 ± 2.7	< 0.001*
JSW, mm	2.9 ± 2.6	3.0 ± 1.5	0.85
CDI, %	0.9 ± 0.2	0.7 ± 0.1	< 0.001*
PTS, °	6.5 ± 4.1	6.5 ± 3.3	0.935
LDTA, °	90.9 ± 3.2	90.6 ± 2.8	0.49
AJLO, °	6.2 ± 4.1	2.7 ± 9.7	< 0.001*

Data are shown as the means with standard deviation or number.

*JKOM* Japanese Knee Osteoarthritis Measure, *VAS* visual analogue scale, *ADL* activities of daily living, *KOOS* Knee Injury and Osteoarthritis Outcome Score, *QOL* Quality of Life, *KSS* Knee Society Score, *TUG* timed up and go test, *SLS* single leg stance, *HAA* hip abduction angle, *HKA* hip-knee-ankle, *FTA* femoro-tibial angle, *WBLR* weight-bearing line ratio, *JSW* joint space width, *PTS* posterior tibia slope, *CDI* Caton-Deschamps index, *MPTA* medial proximal tibial angle, *LDFA* lateral distal femoral angle, *LDTA* lateral distal tibial angle, *JLCA* joint line convergence angle, *KJLO* knee joint line obliquity, *AJLO* ankle joint line obliquity.

### Comparison of the clinical and functional scores and radiographic parameters between the HAA (−) group and the HAA (+) group at 2 years postoperation

Except for the TUG test, no clinical and functional scores were significantly different between the HAA (−) group and the HAA (+) group at 2 years postoperatively. Scores on the TUG test in the HAA (−) group were significantly lower than that in the HAA (+) group (p = 0.011). Regarding the radiological parameters, the HKA, WBLR and KJLO in the HAA (−) group were significantly higher than those in the HAA (+) group (P < 0.001, 0.001 and 0.025). In contrast, the LDFA in the HAA (−) group were significantly lower than those in the HAA (+) group (P < 0.001) (Table 3).

**Table 3:** Comparison of Clinical and functional score and radiographic parameter between HAA (−) group and HAA (+) group at 2 year post-operation.

	HAA (−)-group (n = 52)	HAA (+)-group (n = 38)	*p-*value	*p-*value^#^
Clinical score				
JKOM				
VAS for ADL	16.9 ± 20.9	16.0 ± 21.6	0.84	0.74
Pain and stiffness	5.4 ± 4.9	5.6 ± 5.1	0.85	0.66
ADL	5.2 ± 5.9	5.0 ± 4.9	0.82	0.73
Activities	4.1 ± 4.4	3.2 ± 4.0	0.32	0.53
Health condition	2.0 ± 1.7	1.9 ± 1.8	0.76	0.74
Total	16.8 ± 14.8	15.7 ± 14.0	0.74	0.93
KOOS				
Symptom	81.0 ± 14.8	83.0 ± 16.0	0.54	0.40
Pain	80.5 ± 16.2	80.8 ± 15.6	0.94	0.92
ADL	86.7 ± 13.6	87.6 ± 11.7	0.77	0.67
Sports	60.1 ± 26.4	61.3 ± 25.9	0.83	0.59
QOL	62.8 ± 24.2	65.0 ± 25.6	0.69	0.63
KSS	93.1 ± 5.9	90.6 ± 9.4	0.13	0.18
TUG, s	8.0 ± 1.7	9.0 ± 2.2	0.01*	0.04*
SLS, s	22.0 ± 11.2	21.1 ± 10.7	0.70	0.84
Isometic muscle strength (%BW)				
Quadriceps	137.3 ± 43.5	133.8 ± 40.3	0.70	0.83
Hamstring	75.6 ± 34.7	84.7 ± 31.8	0.20	0.13
Radiological parameters				
HKA, °	3.9 ± 2.9	1.0 ± 4.0	< 0.01*	< 0.01*
LDFA, °	87.7 ± 1.7	89.2 ± 2.5	< 0.01*	0.02*
MPTA, °	93.2 ± 2.9	92.2 ± 3,3	0.13	0.23
WBLR, %	60.3 ± 14.3	50.9 ± 15.2	< 0.01*	< 0.01*
JLCA, °	2.4 ± 1.8	2.1 ± 2.2	0.56	0.82
KJLO, °	3.2 ± 2.7	1.9 ± 2.6	0.03*	0.02*
JSW, mm	2.9 ± 1.5	3.1 ± 1.5	0.44	0.46
CDI, %	0.7 ± 0.1	0.7 ± 0.2	0.99	0.93
PTS, °	6.4 ± 3.2	6.5 ± 3.4	0.84	0.69
LDTA, °	90.7 ± 2.9	90.5 ± 2.6	0.69	0.49
AJLO, °	2.0 ± 3.6	1.5 ± 3.5	0.47	0.43

Data are shown as the means with standard deviation or number.

*JKOM* Japanese Knee Osteoarthritis Measure, *VAS* visual analogue scale, *ADL* activities of daily living, *KOOS* knee injury and osteoarthritis outcome score, *QOL* quality of life, *KSS* Knee Society Score, *TUG* timed up and go test, *SLS* single leg stance, *HAA* hip abduction angle, *HKA* hip-knee-ankle, *FTA* femoro-tibial angle, *WBLR* weight-bearing line ratio, *JSW* joint space width, *PTS* posterior tibia slope, *CDI* Caton-Deschamps index, *MPTA* medial proximal tibial angle, *LDFA* lateral distal femoral angle, *LDTA* lateral distal tibial angle, *JLCA* joint line convergence angle, *KJLO* knee joint line obliquity, *AJLO* ankle joint line obliquity.

^#^Adjusted for base line value (ANCOVA models).

### Correlation between the HAA and clinical and functional scores and radiological parameters at 2 years postoperation

**Figure 1:**
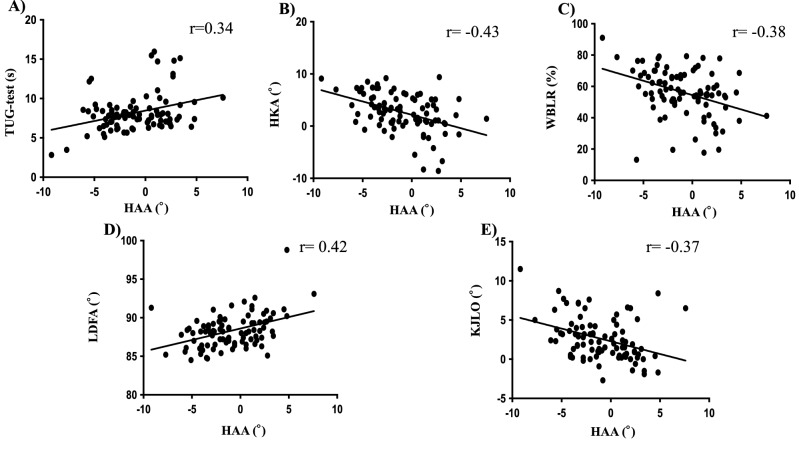
Correlation between HAA and clinical and functional scores and radiological parameters at post-operative 2 year. Correlation between HAA and (**A**) TUG-test, (**B**) HKA, (**C**) WBLR, (**D**) FTA, (**E**) LDFA and (**F**) KJLO.

In clinical and functional scores, TUG scores were positively weakly correlated with the HAA. (r = 0.34, p < 0.001) Regarding the radiological parameters, the LDFA were weakly positively correlated with the HAA (r = 0.42, P < 0.001). In contrast, the HKA, WBLR and KJLO were weakly negatively correlated with the HAA (r = − 0.43, − 0.38 and − 0.37,* P* < 0.001, 0.001 and 0.001) (Table 4 and Fig. 1).

**Table 4:** Correlation between HAA and Clinical and functional scores and radiological parameters.

	r	95% CI	p value
TUG	0.34	0.14 to 0.51	< 0.001
HKA	− 0.43	− 0.59 to − 0.25	< 0.001
LDFA	0.42	0.23 to 0.58	< 0.001
WBLR	− 0.38	− 0.54 to − 0.18	< 0.001
KJLO	− 0.37	− 0.54 to − 0.18	< 0.001

*HAA* hip abduction angle, *HKA* hip-knee-ankle, *FTA* femoro-tibial angle *WBLR* weight-bearing line ratio, *LDFA* lateral distal femoral angle, *KJLO* knee joint line obliquity.

### Transitions in the HAA during follow-up

**Figure 2:**
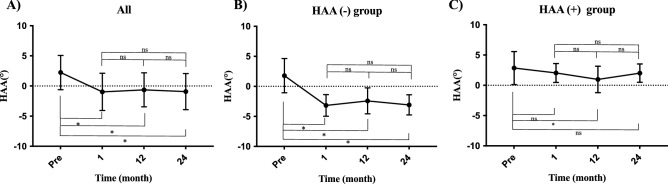
The transition of HAA during follow-up. The transition of HAA in (**A**) all patients, (**B**) HAA (+) group and (**C**) HAA(−) group.

In all patients, the average preoperative HAA was located in the abduction position (HAA = 2.2 ± 2.8). It significantly changed towards the adduction position at 12 months postoperatively (HAA = − 0.6 ± 2.8) and maintained over time at 24 months (HAA = − 0.9 ± 3.0) (Fig. 2A). In the HAA (−) group, the HAA was significantly in the adduction position at postoperative month 12 (HAA = − 2.4 ± 2.1) and was maintained in the adduction position at postoperative month 24 (HAA = − 3.1 ± 1.7) (Fig. 2B). On the other hand, in the HAA (+) group, the HAA was significantly towards a neutral position at 12 months postoperatively (HAA = 0.9 ± 2.2) and changed to the abduction position at 24 months postoperatively (HAA = 2.0 ± 1.5) (Fig. 2C).

## Discussion

Most findings of this study were that postoperative HAA was significantly associated with scores on the TUG test and the HKA, WBLR, LDFA, and KJLO of lower limb alignment. The TUG test and LDFA were weakly positively correlated with the HAA. In contrast, the HKA, WBLR, and KJLO were weakly negatively correlated with the HAA. In addition, the HAA at preoperation was located in the abduction position, changed towards adduction at postoperative year 1 and maintained the neutral position over time.

In a previous study, it was recognized that functional scores improved after OWHTO^14,15^. In addition, walking speed, step length and gait patterns also improved after OWHTO^16–18^. In contrast, Morin et al. reported that gait parameters, including leg stance time, step length, step width and walking speed, do not change after HTO^19^. In this study, functional scores, including JKOM, KOOS and KSS, were improved significantly after OWHTO at 2 years post-treatment. Regarding gait parameters, the SLS test was not changed, and the TUG test was improved. The TUG test was included in daily life (standing up, walking, turning up, sitting down) and indirectly indicated walking speed and step length^20^. Therefore, the results of this study were consistent with those of a former study. Because the latter study was focused on one year postoperatively, it might be different from our results.

There are some studies for lower alignment change after OWHTO^21–23^. However, there are few reports about hip joint alignment compared to the ankle joint of knee adjacent joints. Park et al. reported that changes in KJLO after OWHTO were associated with limb adduction angle after OWHTO, and changes with AJLO were not associated with KJLO^24^. In this study, HAA was negatively correlated with KJLO and was not correlated with AJLO. Our results are consistent with their study. Oh et al. also reported that KJLO was not associated with AJLO^9^. This would be the reason why there is a difference between the ball and socket joint of the hip and the hinged synovial joint of the ankle. Additionally, in this study, the HAA was positively correlated with the HKA and WBLR. In contrast, it was negatively correlated with the LDFA. The results suggested that excessively higher HAAs induced varus recurrence. In addition, HAA was not associated with other parameters, including MPTA, JLCA, JSW, CDI, PTS, LDTA and AJLO. These parameters are not included femoral alignment. Each parameter indicated partially tibial, ankle and intraarticular alignment. Therefore, this result may be suitable.

In surgery for medial osteoarthritis, including total knee arthroplasty (TKA), unicompartmental knee arthroplasty (UKA) and HTO, rehabilitation is targeted for the knee extensor muscle^25,26^. However, hip abduction muscle strength is also important for osteoarthritis patients, and weakness of hip abduction muscle strength is associated with poor outcomes of TKA and UKA^27,28^. Hip abductor muscle weakness induces pelvic tilt and leads to medial compartment loading, which is referred to as the Trendelenburg sign^7^. Thus, hip abductor muscle weakness leads to a higher HAA; on the other hand, sufficient hip abductor muscle leads to a lower HAA. In this study, TUG in the HAA (−) group was significantly lower than that in the HAA (+) group. This result indicated that a higher TUG in the HAA (+) group might be induced by the Trendelenburg gait with hip abductor muscle weakness.

In single-limb standing on walking, a leading medial shift mechanical axis is induced with an increased hip abduction angle of hip abductor muscle weakness, and osteoarthritis progresses^6,29^. In a systematic review reported by Raghava et al., hip muscle strengthening improved knee pain and physical function in osteoarthritis patients^30^. The patients who underwent OWHTO commonly had early osteoarthritis. However, the age, sex and activity of the patients varied. In this study, in cases of a higher HAA at preoperation, the HAA was towards the neutral position at postoperative year 1 and changed again to the abduction position at postoperative month 24. Therefore, they are presumed to be low hip abductor muscle strength in origin. Abductor muscle strength should be considered for a long time if preoperative HAA in OWHTO patients is higher.

This study has several limitations. First, hip abduction muscle strength was not evaluated. Therefore, it is unclear whether hip abduction muscle weakness affects lower HAA. Second, pelvic alignment, such as pelvic tilt, was not measured. Despite the association between scores on the TUG test and the HAA in this study, it remains unclear whether pelvic alignment affects gait parameters. Third, we didn't conduct a multiple testing adjustment. Therefore, the results should be interpreted in an exploratory way.

## Conclusion

This study showed that the postoperative HAA was significantly associated with the TUG test of gait parameters and the HKA, WBLR, LDFA, and KJLO of lower limb alignment. Higher postoperative HAA might induce the varus recurrence and poor outcomes related to the gait parameter.

## Methods

This analysis of prospectively collected data was approved by our hospital ethics committee (IRB No.30-5) and conducted in accordance with the Declaration of Helsinki. Standard informed consent was obtained from all patients participating in this study.

### Patients

**Figure 3:**
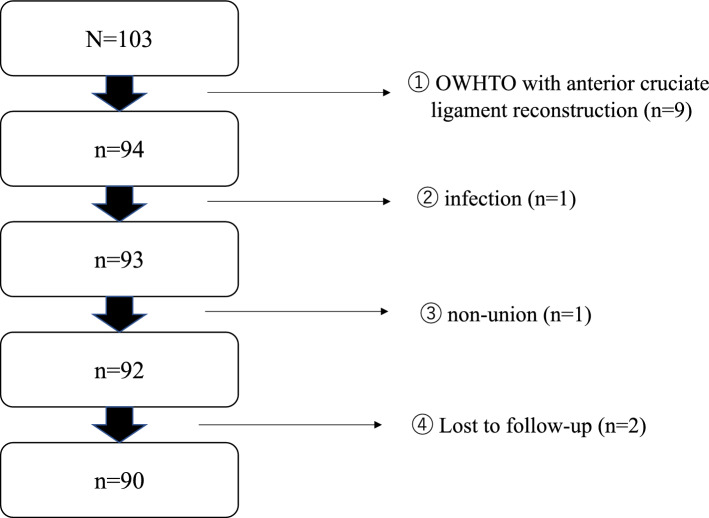
Flowchart of patient selection.

One hundred and three patients who underwent OWHTO for medial compartment osteoarthritis at our hospital from 2011 to 2019 were included in this study. The exclusion criteria were as follows: patients who (1) underwent OWHTO with anterior cruciate ligament reconstruction (n = 9), (2) had an infection (n = 1), (3) had delayed or nonunion within two years (n = 1), and (4) were lost to follow-up (n = 2). A total of 90 patients were ultimately assessed for radiological parameters of lower limb alignment and clinical and functional assessments (Fig. 3). The patients were divided into two groups according to the HAA at 1 month after operation: the HAA (−) group (HAA < 0°) and the HAA (+) group (HAA ≥ 0°).

### Surgical procedure and postoperative rehabilitation

A preoperative plan was conducted using an anteroposterior (AP) long-axis radiography in the supine position and implemented using the medical planning software mediCAD (Hectec GmbH, Germany). Intra-articular chondral and meniscus lesions were evaluated by arthroscopic findings before osteotomy in all patients. The oblique incision was started at the medial tibia, and high tibia osteotomy was conducted towards the proximal tibiofibular ligament. The correction angle was determined to be 62.5% of the % weight bearing line (WBLR) as the defined Fujisawa point using Alignment Rod Methods^12,13^. After placement of an artificial bone graft in the osteotomy site, a TriS Medial HTO Plate System® (Olympus Terumo Biomaterials, Tokyo, Japan) was fixed on the medial tibia with 8 locking screws.

### Radiological parameters

**Figure 4:**
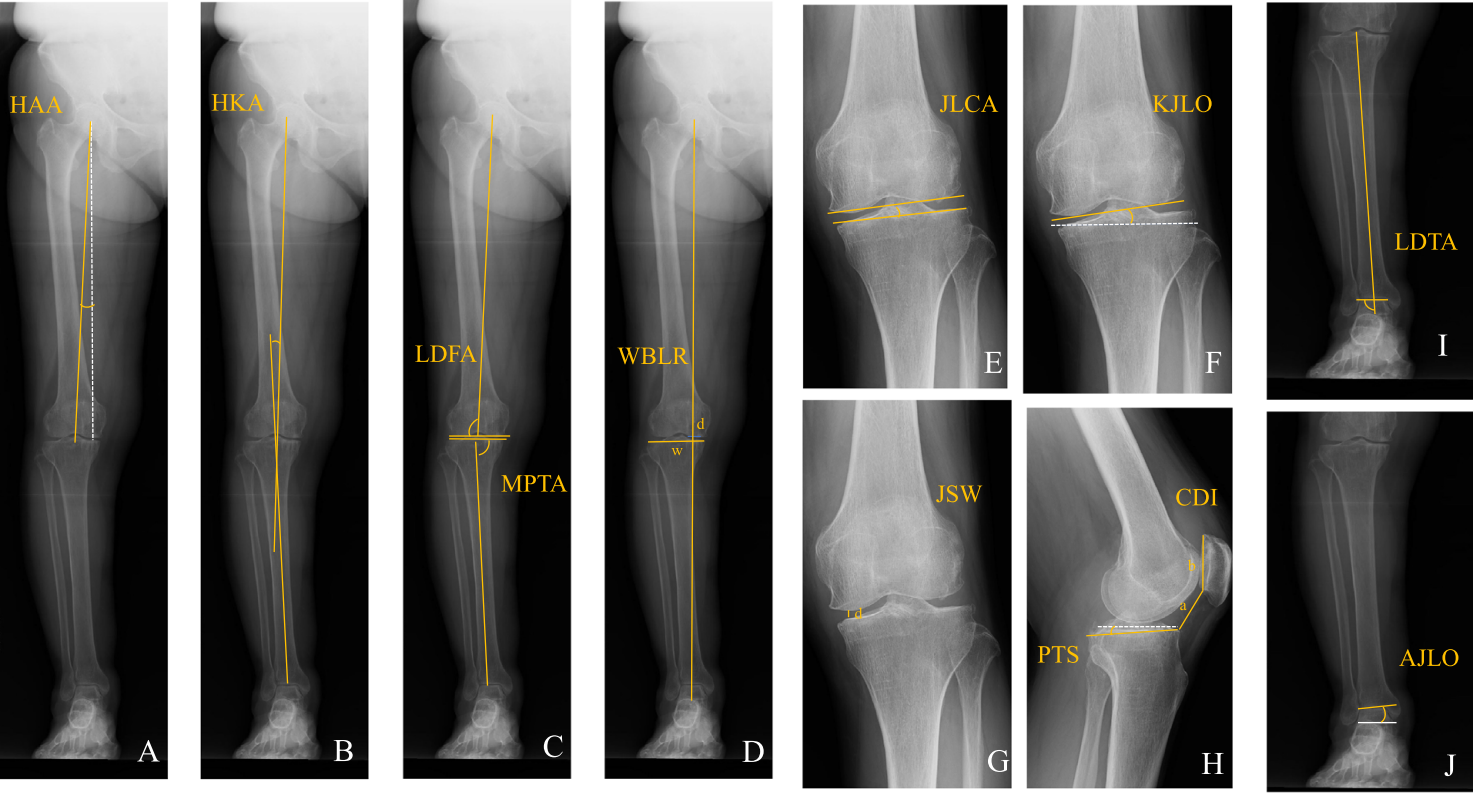
Radiological parameters. (**A**) hip abduction ankle (HAA), (**B**) hip knee ankle (HKA), (**C**) femorotibial angle (FTA), (**D**) lateral distal femoral angle (LDFA) and medial proximal tibial angle (MPTA), (**E**) weight-bearing line ratio (WBLR), (**F**) joint line convergence angle (JLCA), (**G**) knee joint line obliquity (KJLO), (**H**) joint space width (JSW), (**I**) posterior tibia slope (PTS) and Caton-Deschamps index (CDI), (**J**) lateral distal tibial angle (LDTA), (**K**) ankle joint line obliquity (AJLO).

Radiological parameters were assessed on an anteroposterior long-axis radiograph under single-limb standing X-ray and standing lateral radiography preoperatively, one month, 1 year and 2 years postoperatively by two orthopaedic surgeons (Y.K. and M.K.). The intra- and interobserver reliabilities of each measurement were assessed by determining the intraclass correlation coefficient (ICC). Radiological parameters included the hip abduction angle (HAA), hip-knee-ankle angle (HKA), lateral distal femoral angle (LDFA), medial proximal tibial angle (MPTA), weight-bearing line ratio (WBLR), joint line convergence angle (JLCA), knee joint line obliquity (KJLO), joint space width (JSW), posterior tibia slope (PTS), Caton-Deschamps index (CDI), lateral distal tibial angle (LDTA), and ankle joint line obliquity (AJLO). The HAA was defined as the angle between the femoral mechanical axis and vertical axis to the ground with abduction. (Adduction: positive) (Fig. 4A) The HKA was defined as the angle between the mechanical femoral axis and the mechanical tibial axis. (Valgus: positive) (Fig. 4B) The LDFA was defined as the lateral angle between the femoral mechanical axis and the femoral distal joint line. (Fig. 4C) The MPTA was defined as the medial angle between the proximal tibial joint line and the mechanical tibial axis (Fig. 4C). The WBLR was defined as the ratio (%) of horizontal distance to WBL from the medial edge of the tibial plateau, as d/w × 100. (Fig. 4D) The JLCA was defined as the angle between the joint line of the distal femur and the joint line of the proximal tibia. (Lateral opening: positive) (Fig. 4E) The KJLO was defined as the angle between the joint line of the proximal tibia and a parallel line to the ground. (Lateral inclination: positive) (Fig. 4F) The JSW was defined as the distance (mm) between the apex of the medial femoral condyle and medial tibial plateau (Fig. 4 G). The PTS was defined as the angle between a parallel line to the ground and the posterior inclination of the tibial plateau (Fig. 4H). The CDI was defined as the ratio (%) of the distance to the lowest point of the articular cartilage on the patella from the tibial articular edge, as a/b (Fig. 4H). The LDTA was defined as the lateral angle between the joint line of the talus and the tibial mechanical axis (Fig. 4I). The AJLO was defined as the angle between the joint line of the talus and a line parallel to the ground. (Lateral inclination: positive) (Fig. 4J).

### Clinical and functional score

Clinical and functional scores, including the Japanese Knee Osteoarthritis Measure (JKOM), Knee injury and Osteoarthritis Outcome Score (KOOS), Knee Society Score (KSS), timed up and go test (TUG) test, single leg standing (SLS) test and isometric muscle strength (quadriceps and hamstrings), were assessed in outpatients preoperatively and 2 years postoperatively.

### Statistical analysis

The statistical analyses were performed using the GraphPad Prism Biostatistics software program ver 9.4.1. for Mac (GraphPad Software Inc., La Jolla, CA, USA) (URL: https://www.graphpad.com/scientific-software/prism/). To compare the data between the HAA (−) group and the HAA (+) group, we use unpaired t-test and ANCOVA with adjustment of baseline values. The paired t-test was used to assess differences at preoperative and postoperative two years in all patients. The correlation between the HAA and clinical score and radiological parameters was assessed by Pearson’s correlation coefficient. A value of p < 0.05 was considered statistically significant. The data are presented with descriptive statistics as the mean and standard deviation. To assess test–retest reliability measurement, the intraclass correlation coefficients (ICCs) were evaluated X-ray randomly selecting 20 patients. The inter- and intraobserver ICCs of the radiographic data were shown in Supplemental Table S2, indicating excellent reliability.

### Ethical approval

This study was approved by our institution ethics committee (IRB No. 30-5).

### Informed consent

Standard informed consent was obtained from all patients participating in this study.

## Supplementary Information

Supplementary Information 1.

Supplementary Information 2.

## Data Availability

The datasets used and/or analysed during the current study available from the corresponding author on reasonable request.
