# Supplementary material for: Hip abduction angle after open-wedge high tibial osteotomy is associated with the timed up & go test and recurrence of varus alignment
Source: Sci Rep. 2023 Apr 29;13:7047. doi: 10.1038/s41598-023-33481-9 (PMC10148799; doi:10.1038/s41598-023-33481-9)
Supplement: Supplementary file 2 — Supplementary Information 2. [file 41598_2023_33481_MOESM2_ESM.pdf]

Supplemental table 1. Comparison of Clinical and functional score and radiographic parameter between HAA (-) group and HAA (+) group at pre-operation

Clinical score		HAA (-)-group (n=52)	HAA (+)-group (n=38)	*p*-value
JKOM	VAS for ADL	61.1 ± 26.9	59.9 ± 24.4	.83
	Pain and stiffness	16.7 ± 6.6	16.3 ± 6.9	.77
	ADL	12.3 ± 6.3	13.7 ± 7.3	.36
	Activities	7.9 ± 5.4	8.8 ± 6.0	.46
	Health condition	3.5 ± 2.1	3.8 ± 2.1	.51
	Total	40.7 ± 17.5	42.6 ± 18.7	.63
KOOS	Symptom	59.9 ± 18.6	58.7 ± 19.4	.77
	Pain	49.7 ± 19.3	50.1 ± 18.2	.92
	ADL	65.3 ± 16.0	66.7 ± 16.6	.68
	Sports	31.5 ± 20.2	28.9 ± 13.8	.56
	QOL	29.6 ± 17.0	27.0 ± 13.8	.41
KSS		65.3 ± 6.6	67.2 ± 5.9	.17
TUG, s		10.4 ± 2.6	9.8 ± 2.5	.18
SLS, s		18.4 ± 12.2	20.1 ± 11.5	.49
Isometic muscle strength (%BW)	Quadriceps	117.0 ± 48.5	102.7 ± 44.5	.15
	Hamstring	52.9 ± 24.4	53.1 ± 31.7	.98

Radiological parameters	HAA (-)-group (n=52)	HAA (+)-group (n=38)	*p*-value
HAA, °	1.8 ± 2.9	2.9 ± 2.7	.09
HKA, °	-6.2 ± 3.3	-8.5 ± 3.4	< .001*
LDFA, °	88.5 ± 1.9	89.1 ± 2.0	.16
MPTA, °	85.2 ± 2.2	84.4 ± 2.9	.12
WBLR, %	19.0 ± 10.7	13.1 ± 14.3	.03*

JLCA, °	3.1 ± 1.9	2.7 ± 2.2	.32
KJLO, °	0.4 ± 3.2	0.3 ± 3.1	.83
JSW, mm	2.9 ± 3.0	3.0 ± 2.0	.81
CDI, %	0.9 ± 0.1	0.9 ± 0.2	.94
PTS, °	6.1 ± 3.4	7.0 ± 4.9	.34
LDTA, °	90.8 ± 3.0	91.0 ± 3.5	.72
AJLO, °	5.9 ± 3.7	6.6 ± 4.5	.43

Data are shown as the means with standard deviation or number.

※HAA: Hip abduction angle, HKA: Hip-knee-ankle, FTA: Femoro-tibial angle

WBLR: Weight-bearing line ratio, JSW: Joint space width, PTS: Posterior tibia slope, CDI: Caton-Deschamps index

MPTA: Medial proximal tibial angle, LDFA: Lateral distal femoral angle, LDTA: Lateral distal tibial angle

JLCA: Joint line convergence angle, KJLO: Knee joint line obliquity, AJLO: Ankle joint line obliquity

Suppleental table 2. ICC for Inter- and Intraobserver agreement of the radiographic data

	Interobserver ICC	Intraobserver ICC
HAA, °	0.880	0.936
HKA, °	0.858	0.924
LDFA, °	0.797	0.887
MPTA, °	0.885	0.939
WBLR, %	0.921	0.952
JLCA, °	0.740	0.851
KJLO, °	0.862	0.926
JSW, mm	0.803	0.826
CDI, %	0.721	0.838
PTS, °	0.771	0.871
LDTA, °	0.728	0.843
AJLO, °	0.819	0.900
